# Schmallenberg Virus Infection among Red Deer, France, 2010–2012

**DOI:** 10.3201/eid2001.130411

**Published:** 2014-01

**Authors:** Eve Laloy, Emmanuel Bréard, Corinne Sailleau, Cyril Viarouge, Alexandra Desprat, Stéphan Zientara, François Klein, Jean Hars, Sophie Rossi

**Affiliations:** Ecole Nationale Vétérinaire d’Alfort, Maisons-Alfort, France (E. Laloy);; French Agency for Food Environmental and Occupational Health and Safety, Maisons-Alfort (E. Breard, C. Sailleau, C. Viarouge, A. Desprat, S. Zientara);; French Wildlife and Hunting Agency, Bar-le-Duc, France (F. Klein);; French Wildlife and Hunting Agency, St Benoist, France (J. Hars, S. Rossi)

**Keywords:** Vector-borne disease, Orthobunyavirus, SBV, wildlife, serology, ELISA, viruses, Schmallenberg virus, France

## Abstract

Schmallenberg virus infection is emerging in European domestic and wild ruminants. We investigated the serologic status of 9 red deer populations to describe virus spread from September 2010 through March 2012 among wildlife in France. Deer in 7 populations exhibited seropositivity, with an average seroprevalence of 20%.

In summer and fall 2011, an unidentified disease was reported in dairy cattle in Germany and the Netherlands, causing decreased milk production, fever, and diarrhea (*1*,*2*). The virus associated with these clinical signs was identified as a new member of the genus *Orthobunyavirus* of the Simbu serogroup and named Schmallenberg virus (SBV) (*2*). This virus was later associated with abortions and congenital malformations in calves, lambs, and kids in several European countries (*3*). Serologic testing among wild cervids in Belgium revealed antibodies against Schmallenberg virus in roe deer (*Capreolus capreolus*) and red deer (*Cervus elaphus*) (*4*). Seroprevalence was already high (27% on average) in wild cervids in October 2011 in Belgium, suggesting that the virus began circulating months earlier (before August 2011). It has recently been shown that SBV had already circulated in *Culicoides* vectors in Belgium during August and September 2011 (*5*). Although SBV has been closely monitored among domestic ruminants in France, suggesting that clinical cases and antibodies appeared almost at the same time during 2011–2012 (*6*), little is known about the geographic spread of SBV in wildlife. To correct this lack of data, we conducted a serologic study using serum specimens collected from red deer in different regions in France.

## The Study

Blood samples from 502 red deer, which had been either killed by gunshot or captured, were collected within 9 French departments (administrative units) during 1 or 2 sampling seasons (i.e., during September 2010–January 2011 and September 2011–March 2012). The serum specimens were first screened by using an SBV indirect ELISA (i-ELISA) that was previously validated for the serum specimens from cattle, sheep, and goats (ELISA ID Screen Schmallenberg Virus Indirect, Bicupule; ID Vet, Montpellier, France) (*7*). The results were expressed as S/P values using the cutoff recommended for domestic species (S/P = [optical density sample (S)/optical density positive control (P)] × 100); S/P<60%, negative; S/P>70%, positive; and S/P 60–70%, doubtful result). Serum specimens were also tested with a new competitive ELISA (c-ELISA; ELISA ID Screen Schmallenberg Virus Competitive; ID Vet). Positive results by c-ELISA corresponded to a percentage of inhibition (PI) <50, doubtful result if 40>PI≤50, and negative when PI >50. The antigen used in both c-ELISA and i-ELISA is the same N recombinant protein. A subset of samples were also subjected to a seroneutralization test (SNT) as described (*7*).

Of 502 serum specimens, 492 could be tested by using i-ELISA and 486 by using c-ELISA. The 2 ELISA methods exhibited a 92% match (449/486). Because our samples (taken from dead animals in nonsterile conditions) generated bacterial contamination or cytotoxicity, conclusive SNT results were available from 114 animals only: 64 samples with positive or doubtful i-ELISA results and 50 samples (S/P>20) with negative i-ELISA results. A large part of the serum specimens that were positive or doubtful by ELISA methods were also positive for SBV by SNT, suggesting a good specificity of both methods, though slightly better for c-ELISA than for i-ELISA (Table 1). Many serum specimens that tested negative by i-ELISA or c-ELISA (all collected during 2011–2012) were positive by SNT (Table 1). Even though the c-ELISA kit appeared slightly more sensitive than the i-ELISA kit, these results suggest that SNT is the most sensitive technique for detecting antibodies against SBV in a recently infected population of red deer. These results are consistent with the fact that SNT and c-ELISA are able to detect IgG and IgM, whereas i-ELISA detects only IgG that appears after the IgM adaptive response (E. Breard, pers. comm.). Considering the performance of serologic methods in that study, seroprevalence was finally estimated as the proportion of positive or doubtful serum specimens by using the c-ELISA kit.

**Table 1:** Serologic results for red deer serum samples tested for Schmallenberg virus with SNT, i-ELISA, and c-ELISA*

ELISA method	SNT method				
	SNT positive, n = 97			SNT negative, n = 17	
Positive or doubtful	Negative		Positive or doubtful	Negative
i-ELISA	57	40		7	10
c-ELISA	67	30		6	11
i-ELISA and c-ELISA	49	22		6	9

*SNT, seroneutralization test; i-ELISA, indirect ELISA; c-ELISA, competitive ELISA.

The number of samples collected in each department, the proportion of positive specimens, and the date of first observation of seropositive result are indicated in Table 2. The 56 serum specimens collected during September 2010–February 2011 in northeastern and southwestern France (Bas-Rhin and Pyrenees-Atlantiques departments) were negative by both ELISAs. From September 2011 through March 2012, a total of 7 of 9 departments exhibited at least 1 seropositive specimen by c-ELISA. Among these 7 departments, the average seroprevalence was 20% (95% CI 16%–24%), with significant variations between the 7 departments exhibiting seropositive results (8%–49%) (χ^2^ = 67.4, df = 6, p<0.001). Seroprevalence was not influenced by the animal’s age, suggesting an equal exposure of fawns born in 2011 and older animals (χ^2^ = 0.16, df = 2, p = 0.92). It is thus likely that SBV had not spread to France before red deer in France gave birth to young in spring 2011 (mid-May to early June) (*8*). Seroprevalence varied significantly with the period (χ^2^ = 25.0, df = 2, p<0.001). On average, seroprevalence was higher in December 2011–January 2012 (31%; 95% CI 25%–37%]) compared with September–November 2011 (7%; 95% CI 3%–12%) or February–March 2011 (14%; 95% CI 7%–22%).

**Table 2:** Results of c-ELISA and indication of first seropositive result for Schmallenberg virus in red deer by department, France, 2010–2012*

Department	Average distance to Meurthe-et-Moselle department, km†	No. positive samples/ no. tested in 2010–2011 (95% CI, %)	2011–2012		First positive result, 2011
			No. positive samples/no. tested	Mean prevalence, % (95% CI)	
Moselle	46		4/26	15 (2–29)‡	Nov 5
Haute-Marne	102		26/53	49 (36–63)‡	Nov 12
Bas-Rhin	103	0/41 (<7)§	8/53	15 (6–25)‡	Oct 25
Côte d’Or	184		11/37	30 (15–45)‡	Dec 3
Oise	282		26/69	38 (26–49)‡	Dec 19
Loir-et-Cher	375		11/132	8 (4–13)‡	Nov 25
Hautes-Pyrénées	789	0/14 (<19)§	1/12	8 (0–30)‡	Dec 10
Corsica	749		0/23	0 (<12)§	
Pyrénées Atlantiques	815		0/26	0 (<11)§	

*c-ELISA, competitive ELISA. †Department where first domestic clinical cases were reported, January 25, 2012. ‡95% CIs were estimated by assessing a binomial distribution of seroprevalence. §Upper value of the 95% CI was estimated according a hypergeometric distribution of the risk to detect at least 1 positive result (p = 1–exp(Ln(0,05)/N), with N being the sample size).

These results suggest that SBV was actively circulating during fall 2011 until mid-November or early December. In agreement with the findings of Linden et al. for Belgium (*4*), we consider that the mild temperature observed in France in fall 2011 may have favored a late activity of vectors (*9*). The date of first occurrence of seropositive red deer and the seroprevalence observed in each department (Table 2) were not strictly dependent on the distance from the Meurthe-et-Moselle department where the first domestic case (congenital form) had been confirmed on January 25, 2012 (*10*) (Figure). This result possibly arose because of uncontrolled variations in the sampling dates of red deer between the 9 departments and still unknown factors associated with SBV spread. Nevertheless, most of the departments that exhibited seropositive red deer from September 2011 to March 2012 had also reported clinical cases in domestic flocks during January–March 2012 (Figure).

**Figure:**
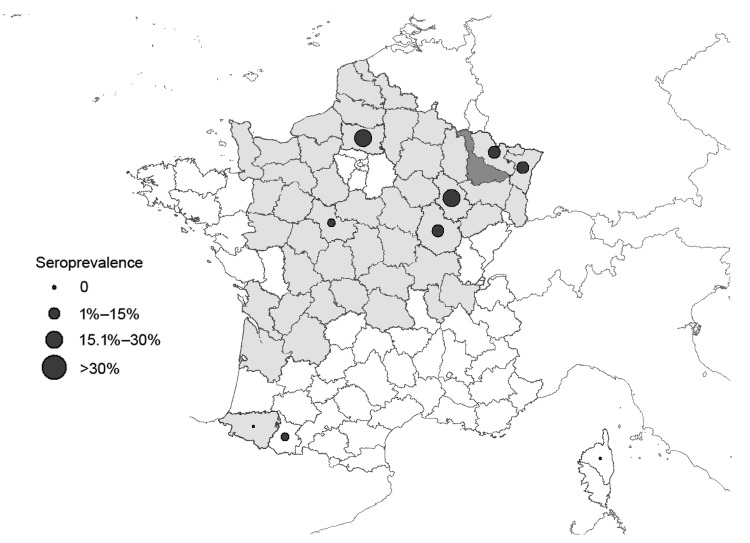
Sites where serum samples were obtained from red deer (9 departments), showing average seroprevalence for Schmallenberg virus, France, 2010–2012. Dark gray shading indicates Meurthe-et-Moselle department, where the first domestic case was found; light gray shading indicates departments where clinical cases were found during January–March 2012; and white indicates departments where no clinical cases occurred during January–March 2012.

In southwestern France (near the Pyrénées Mountains), a red deer seropositive for SBV was observed in the Hautes-Pyrénées department, whereas congenital clinical cases of SBV infection in domestic livestock (congenital malformations on kids) had been reported by March 30, 2012, in the neighboring Pyrénées-Atlantiques department (E. Bréard, pers. comm.) (Figure). These results suggest similar spread of SBV among red deer and domestic livestock during fall 2011 at the department level. In 2012, no evidence of abortions or malformations was reported in red deer or other native wildlife ruminant species within the populations monitored by wildlife biologists or zoo veterinarians in France (S. Rossi, A. Decors, A. Lécu, pers. comm.). However, specific studies exploring the effect of SBV on the reproductive success of wild species are still lacking.

## Conclusions

This study provides a preliminary view of SBV spread among wild cervids in France during 2010–2012. Our data suggest that SBV spread quickly from northeastern to southwestern France (≈800 km) between October and December 2011. Our data also show the match of SBV spread among red deer and domestic flocks at the level of the department and highlight the perspective that red deer can be a sentinel of SBV spread for livestock. We also pinpointed the relevance of new competition ELISA for improving SBV surveillance in wildlife species, even though SNT remained the most reliable assay for SBV antibody detection in red deer. Further studies that encompass several years and include a larger number of species and localities would help provide a more complete picture of virus spread and risk factors in wildlife (*11*).
